# The Cerebellum and Premenstrual Dysphoric Disorder

**DOI:** 10.3934/Neuroscience.2014.2.120

**Published:** 2014-07-30

**Authors:** Andrea J. Rapkin, Steven M. Berman, Edythe D. London

**Affiliations:** 1 USA David Geffen School of Medicine at UCLA, Box 951740, 27-139 CHS, Los Angeles, CA 90095, USA; 2 Center for Addictive Behaviors, Department of Psychiatry and Biobehavioral Sciences, UCLA, 90095 USA; 3 Department of Psychiatry and Biobehavioral Sciences, Department of Molecular and Medical Pharmacology, and Brain Research Institute, UCLA, 90095 USA

**Keywords:** premenstrual dysphoric disorder, premenstrual syndrome, cerebellum, neuroimaging, brain aging, emotional disorders, leptin

## Abstract

The cerebellum constitutes ten percent of brain volume and contains the majority of brain neurons. Although it was historically viewed primarily as processing motoric computations, current evidence supports a more comprehensive role, where cerebro-cerebellar feedback loops also modulate various forms of cognitive and affective processing. Here we present evidence for a role of the cerebellum in premenstrual dysphoric disorder (PMDD), which is characterized by severe negative mood symptoms during the luteal phase of the menstrual cycle. Although a link between menstruation and cyclical dysphoria has long been recognized, neuroscientific investigations of this common disorder have only recently been explored. This article reviews functional and structural brain imaging studies of PMDD and the similar but less well defined condition of premenstrual syndrome (PMS). The most consistent findings are that women with premenstrual dysphoria exhibit greater relative activity than other women in the dorsolateral prefrontal cortex and posterior lobules VI and VII of the neocerebellum. Since both brain areas have been implicated in emotional processing and mood disorders, working memory and executive functions, this greater activity probably represents coactivation within a cerebro-cerebellar feedback loop regulating emotional and cognitive processing. Some of the evidence suggests that increased activity within this circuit may preserve cerebellar structure during aging, and possible mechanisms and implications of this finding are discussed.

## 1. Introduction

Premenstrual dysphoric disorder (PMDD) affects 2%–5% of women in their reproductive years, and is characterized by affective, behavioral and somatic symptoms that recur monthly in the luteal phase of the menstrual cycle and resolve during menstruation. The affective symptoms, which are the most debilitating in the syndrome, include marked irritability or anger, depressed mood, anxiety, tension, mood lability, difficulty concentrating, and feeling overwhelmed or out of control [1,2].

Behavioral and somatic symptoms contribute to impairment, and can include decreased interest, sleep and appetite disturbances, fatigue, lethargy, poor concentration, swelling, and pain [1]. Preceding the classification of PMDD in the Diagnostic and Statistical Manual of Mental Disorders [1], a syndrome characterized by the occurrence of these symptoms was classified as severe premenstrual syndrome (PMS). PMS is now considered to be a similar but imprecisely defined category of premenstrual disorder [3]. Although a link between menstruation and cyclical dysphoria had been recognized since the time of Hippocrates [4], the connection has only recently been explored from the perspective of neuroscience [2].

## 2. Why might the cerebellum be involved in PMDD?

While constituting only 10% of brain volume, the cerebellum contains the majority of neurons in the brain, about 3.6 times the number in the cerebral cortex. This ratio of cerebellar to neocortical neurons is conserved across mammalian species, indicating a co-evolution that is consistent with a coordinated integrative functioning of the two structures [5]. Moreover, reports of crossed human cerebrocerebellar atrophy are thought to reflect cerebellar degeneration after disconnection with the contralateral cerebral hemisphere [6], suggesting that dynamic communication determines the ratio of cerebellar to cerebral neurons in postnatal development. The cerebellum is ideally suited to fine-tune all forms of computation through reverberating cerebro-cerebellar feedback loops [7]. There is a densely packed, highly vascularized and homogeneous layered neural architecture in the cerebellum that connects to all regions of the cerebral cortex through both the pons and thalamus, and while the cerebral cortex is about 50% grey matter, the cerebellar cortex is 80% gray matter.

Consistent with this link to the cerebral cortex, the historical view of the cerebellum as a motor machine has been expanded to encompass a more comprehensive role, including the modulation of cognitive and affective behavior. Based in part on observations of Harry Harlow’s socially-deprived monkeys [8], James Prescott proposed that cerebellar hyperactivity produced by sensory deprivation accounted for the emotional disorders resulting from isolation rearing, and postulated a major role for the cerebellum in emotional regulation [9].

Early clinical support for this idea came from studies in which the deep cerebellar regions were stimulated in patients with behavioral disorders, including psychosis, aggression, and epilepsy [10–12]. Stimulation of the posterior vermis and associated cerebellar regions improved alertness, aggression, anger and depression. This work led to the identification of these regions as a “pacemaker” and clinical target for the limbic modulation of affect [11], and the discovery of disordered affect in patients with prior damage to these regions [13–15].

In 1998, damage to the posterior but not the anterior cerebellar lobe was shown to result in a cerebellar cognitive affective syndrome (CCAS), characterized by executive, visuospatial, linguistic and affective deficits [16]. Further clinical evaluation revealed deficits within five domains: attentional control, emotional control, social skills, autism spectrum behaviors, and psychosis spectrum behaviors [17]. In the same way that cerebellar damage produces both hypometria (undershoot) and hypermetria (overshoot) of motor behavior, both flattened and excessive emotional responses were noted in some CCAS patients within each of the five domains, including virtually all of the affective symptoms associated with PMDD.

The Dysmetria of Thought Theory [18,19] postulates that the anatomical organization of the cerebellar cortex subserves congruent and simultaneous modulation of diverse streams of information in functional domains, as determined by the anatomic locations of the cerebellar and extracerebellar regions constituting each loop. This modulation allows for automated integration of internal representations with external stimuli, serving as an oscillation dampener that optimizes self-generated responses according to context. This idea was consistent with the results of studies in which anatomically different but consistent cerebellar activations were recorded during motor and non-motor versions of cognitive tasks [20].

The prediction of topographic organization of function within the cerebellum was evaluated in a 2009 meta-analysis of neuroimaging studies reporting cerebellar activation during tasks that could be considered to involve one primary category of processing [21]. Emotional processing was localized using data from nine publications that in total assessed 149 normal subjects who were engaged in viewing emotional pictures or facial expressions, identifying the emotion of a speaker, or adopting the emotion of a pictured face. These tasks produced consistent activations in posterior cerebellar lobules VI and VII, both in the vermis and cerebellar hemispheres. This anatomical pattern discriminated activation produced during emotional tasks from that which was produced during performance of motor and somatosensory tasks. However, lobules VI and VII were also activated during linguistic, working memory and executive function tasks, suggesting extensive anatomical overlap between emotional and cognitive processing [21]. Lobule VI was also activated during spatial tasks, and the vermis and fastigial nucleus have been implicated in both vestibular and emotional balance [22]. These observations support the view that some cerebellar regions have multiple functions.

A study of neural response to emotional faces, measured with functional magnetic resonance imaging (fMRI), found activation in lobules VI, VII and the posterior vermis of the cerebellum [23]. Because most of these effects were evoked by faces portraying negative emotions, concomitant with activation in mirror neuron domains, such as the amygdala and insula, the authors suggested that the cerebellum participates in control of negative emotions and goal-directed behavior. Alternatively, faces portraying fear and anger may be more activating than those that are neutral or happy. Another study had results that could be interpreted similarly. Non-invasive transcranial direct current anodal and cathodal stimulation (tDCS) over the cerebellum, but not over prefrontal cortex, enhanced sensory processing of negative, but not positive or neutral facial expressions [24]. The consensus of a panel of 17 experts was that stimulation of the cerebellum, either with tDCS or transcranial magnetic stimulation (TMS), can effectively influence cerebellar functions in cognitive and affective, as well as motor domains [25].

In another consensus paper, 16 experts on cerebellar physiology agreed that the cerebellum serves as a “supervised learning machine” that automates sequences of behaviors, thoughts and affects through the evolutionary adaptation and expansion of more primitive motoric internal control models [22]. The authors postulated different contributions of the cerebellum to cognition. These included modulation of timing, anticipation, the appreciation of cause-and-effect relationships, temporal sequencing of verbal working memory, and the evolution of the skillful manipulation of ideas and language, including generative grammar [22]. The view of the neocortex as the sole or primary driver of behavior was no longer deemed tenable and available evidence required conceptualization of the brain as an ensemble, with a central role for cerebro-cerebellar integration [22].

This view fits well with a modification of earlier hypotheses of cognitive aging (reviewed by West [26]), which proposes that dual measures of prefrontal and cerebellar degeneration and the resulting disruptions of fronto-cerebellar control loops better explain age-related changes in processing speed, variability, automaticity, and higher level cognition than frontal measures alone [27]. Support for this view has subsequently accrued from evidence that frontal and cerebellar cortices (along with anterior insula) exhibit the most pronounced age-related gray-matter loss of all brain regions studied [28], and that age-related changes in cerebellar structure are significantly correlated with changes in associative learning [29], processing speed [30–32] and intelligence in the elderly [33].

Using source-based morphometry, it was shown that age-related changes in processing speed were best predicted by structural changes both in the frontal lobe and in cerebellar networks [32]. The predictive power of the frontal network, however, was lost after controlling for cerebral small vessel disease, but that of the cerebellar network was not [32]. In a study using voxel-based morphometry, cerebellar gray matter volume predicted general cognitive ability of 228 older adults even after controlling for total intracranial volume and frontal gray- and white-matter volumes [33]. Combined with results of brain imaging studies, detailed below, these findings have implications for the role of the cerebellum in PMDD.

## 3. Neuroimaging studies of PMDD and related disorders

Over the past 15 years, a variety of brain imaging techniques have been applied to the study of PMDD and related disorders (Table 1). The techniques used include magnetic resonance spectroscopy (MRS), single photon emission computed tomography, positron emission tomography, and structural and functional magnetic resonance imaging. Most studies have been of small sample size, and did not hypothesize or specifically investigate a role for the cerebellum. We will show, however, that evidence for cerebellar involvement emerged nonetheless from the extant brain imaging studies, considered as a whole.

### 3.1. Magnetic resonance spectroscopy

MRS has been used in relevant preliminary studies of brain metabolites, and the neurotransmitters γ-aminobutyric acid (GABA) and glutamate. In the first study [34], MRS was used to measure phase-related changes in brain metabolites in the frontal and parietal cortex ― specifically ratios of N-acetyl-aspartate, choline and myo-inositol to creatine. These ratios are thought to provide information on membrane phospholipid metabolism, high-energy phosphate metabolism and intracellular pH in affective disorders [35,36]. The myo-inositol/creatine ratio exhibited a nonsignificant trend toward higher levels in the PMDD patients in the luteal phase compared with the follicular phase of the menstrual cycle, but differences between the PMDD and control groups were not significant. The results suggested that the menstrual cycle phase-dependent changes in ovarian hormonal concentrations may affect brain chemistry differently in women with or without PMDD.

Another small MRS study quantified GABA, a neurotransmitter that had been implicated in anxiety and depressive disorders, including PMDD [39]. Within midline occipital cortex, 9 women with PMDD exhibited a smaller GABA signal than 14 controls during the asymptomatic follicular phase, but not during the symptomatic luteal phase of the menstrual cycle. Other brain regions were not assessed. This preliminary finding implied disturbances in cortical GABA in women with PMDD.

In another MRS study, there was a decline in glutamate levels from the follicular to the luteal phase, measured as the ratio of glutamate to creatinine, and the change was equivalent in 12 women with and 13 women without PMDD [42]. The findings could imply, as suggested by the authors, that women with PMDD have an exaggerated behavioral sensitivity to the normal phase-related alterations in glutamate, or alternatively that glutamate is not involved in PMDD.

### 3.2. Positron emission tomography (PET) studies of serotonin-system markers

Prompted by the clinical response of PMDD patients to selective serotonin reuptake inhibitors [50–52], positron emission tomography (PET) studies of premenstrual dysphoria explored the role of serotonergic activity. One study quantified serotonin 5-HT1a receptor binding in six regions of interest, which were chosen on the basis of high density of serotonin 5-HT1a receptors: dorsolateral prefrontal cortex, orbitofrontal cortex, anterior cingulate cortex, amygdala, hippocampus and dorsal raphe [40]. In the dorsal raphe, 5-HT1a receptor binding increased from the follicular to the luteal phase more in control participants than in women with PMDD, consistent with a possible disorder of serotonergic function in women with PMDD. The cerebellum served as the reference region rather than a site of primary investigation.

A second PET study quantified brain trapping of ^11^C-labeled 5-hydroxytryptophan, the immediate biosynthetic precursor of serotonin, in whole brain, dorsolateral and medial prefrontal cortices, putamen and caudate in women with PMDD [41]. Trapping of radiolabeled 5-hydroxytryptophan is an indication of the activity of the enzyme, L-amino acid decarboxylase, critical for the synthesis of serotonin. Although there was no control group, changes from the follicular to the symptomatic luteal phase in self-rated negative mood symptoms of PMDD were correlated with concomitant decreases in trapping of 5-hydroxytryptophan in whole brain and most of the assessed regions of interest, particularly the dorsolateral prefrontal cortex. Correlations were much weaker for physical symptoms of PMDD, supporting a role for serotonin synthesis in PMDD. Here again, the cerebellum was not assessed *per se*, precluding any conclusions regarding an effect of serotonin in that structure on symptoms of PMDD.

In sum, these studies provide some support for GABAergic and serotonergic involvement in PMDD, but because the findings came from small samples and require replication, further work in this area is warranted. As the cerebellum either was not assessed or was used as a reference region, its role in PMDD cannot be evaluated from these studies.

### 3.3. SPECT/PET studies of cerebral blood flow and glucose metabolism

We group these studies together because cerebral blood flow is tightly coupled to glucose metabolism, both reflecting local brain function [53]. The first neuroimaging study of premenstrual dysphoria [37] used single photon emission computed tomography (SPECT) with the [^99m^Tc]-hexamethylpropylene amine oxime (HMPAO). Changes in regional cerebral blood flow (rCBF) from the asymptomatic follicular phase to the symptomatic late luteal phase were quantified in seven women with PMS and matched healthy controls. Regions of interest were drawn on the images to measure radioactivity in frontal, temporal and a region including both parietal and occipital cortices, the thalamus, and the basal ganglia of each hemisphere. Radioactivity in the regions was normalized to counts in the cerebellum. There were marked decreases in this normalized measure of rCBF in the temporal lobes on the premenstrual compared to the postmenstrual scan in PMS patients but not in controls, and the temporal lobe rCBF changes were correlated with the changes in self-reports of depressive symptoms in the PMS patients [37].

More recently, we employed PET with [^18^F]fluorodeoxyglucose (FDG) to assess regional brain glucose metabolism and self-rated mood during performance of an affectively neutral vigilance task in women with PMDD and healthy controls [54]. After whole-brain correction, there was a significant difference between the groups in relative regional glucose metabolism only in the cerebellum. A large cluster of voxels in the cerebellar vermis, fastigial nuclei, and lobule VI of the cerebellar hemispheres showed an increase in glucose metabolism comparing the follicular to the luteal phase in PMDD patients, but not controls. In addition, the magnitude of increased activity was correlated with concurrent worsening of mood in PMDD patients. Although the results implicate activation of the “emotional” cerebellum in the genesis of PMDD symptoms, the data cannot determine whether the activation contributes to worsening mood, or is a compensatory response to worsening mood. The cerebellum is rich in GABA_A_ receptors. Because exposure to progesterone, the dominant hormone in the luteal phase of the cycle, alters the configuration of the subunits of these receptors and produces anxiety-like behavior in rodent models of PMDD, the results were interpreted as consistent with a view proposed by previous investigators that a deficiency in mechanisms regulating cycling of subunits of GABA_A_ receptors may be central to PMDD pathophysiology [55].

In 1998, ovarian suppression induced by a gonadotropin-releasing hormone analog was used to show that PMS symptoms are an abnormal response to normal hormonal changes [56]. The investigators who reported this finding recently applied the same design in a multimodal imaging study of PMDD [47]. While participants performed a working memory task (2-back minus 0-back), rCBF was measured using [^15^O]H_2_O and PET while the fMRI blood-oxygen level dependent (BOLD) signal was also quantified. The paradigm was repeated during ovarian suppression, and two subsequent conditions in which estrogen (mid follicular levels) or progesterone (mid-luteal levels), randomized for order, were replaced. As before [56], premenstrual symptoms remitted during ovarian suppression and resumed when either estrogen or progesterone were restored, but healthy controls reported no adverse symptoms with hormonal suppression or restoration.

Across all three hormone conditions, working memory generated greater PET activation in women with PMDD than controls in nine regional clusters. The largest cluster, comprising 53% of the voxels in all of the nine clusters, was in the cerebellum. Notably, the cerebellar effect was stronger in the PET assessment. Task-related activation in PMDD patients was correlated with a measure of psychosocial/somatic impairment, serving as an index of disease severity, in 10 clusters that overlapped with the areas of greater activation in PMDD patients. Frontal cortex and cerebellum represented, respectively, 62% and 32% of the voxels where impairment was correlated with activation related to working memory. PMDD duration and age of onset also were correlated with task-related activation and the extent to which activation was greater in PMDD patients than controls bilaterally in both structures (see Figure 3) [47]. All five significant clusters in the cerebellum were confined to lobules VI and VII, the “emotional” areas of the cerebellum [21]. In sum, despite representing only 10% of brain volume, the cerebellum was the location of the strongest and largest abnormality in activation related to working memory in PMDD and a disproportionately large number of the voxels where activation was associated with PMDD severity and duration.

It is unfortunate that in the initial SPECT study, the cerebellum was chosen as the reference region, preventing evaluation of abnormalities in cerebellar rCBF, and possibly influencing the measures of perfusion in other structures [37]. The menstrual cycle-related decrease in temporal cortex blood flow in symptomatic PMS patients, which was the main finding of that study [37], was not well supported by the other two studies, although one of them found small clusters within the temporal lobes that accounted for 1% of the voxels showing greater PET activation in PMDD summed across symptomatic and asymptomatic conditions and 2% of the voxels where activation was correlated with psychosocial/somatic impairment [47]. In contrast, both of the aforementioned studies that assessed the cerebellum found that greater activation within this structure represented the most prominent difference in the brains of women with PMDD as compared to healthy controls [47,54]. In those studies, cerebellar activation in PMDD also was correlated with a measure of symptom severity and localized to the “emotional” parts of the cerebellum [21], although the effects were confined to the symptomatic luteal phase in the FDG PET study [54] and were assessed across the one asymptomatic and the two symptomatic hormone conditions in the O-15 PET study [47].

In summary, SPECT/PET studies of PMDD point to differences in cerebral blood flow and glucose metabolism as functional indices in women with PMDD. The evidence is that the differences are predominantly in the cerebellum, and specifically in anatomical regions associated with emotional processing and control.

### 3.4. Functional MRI studies

The first fMRI study of premenstrual dysphoria presented emotionally positive, negative and neutral words to women with PMDD and healthy controls, using a Go/NoGo paradigm designed to probe emotional and inhibitory processing [43]. Analyses were applied to medial and lateral orbitofrontal cortices, amygdala and ventral striatum, with age of the participant modeled as a covariate of no interest. Lower performance accuracy in the luteal than the follicular phase by women with PMDD was interpreted as resulting from deficient impulse control via prefrontal “top-down” modulation of the limbic system. A significant menstrual phase by group interaction supported this finding: in the late luteal phase, negative words elicited greater activity in medial orbitofrontal cortex and less activity in lateral orbitofrontal cortex and amygdala of healthy women than in the follicular phase, whereas women with PMDD showed the opposite patterns. Although the authors performed preliminary analysis in brain regions outside the areas of interest, the effects in these regions, including the cerebellum, did not survive correction for whole-brain search volume.

Another group used a Go/NoGo task paired with fMRI to explore brain activity during response inhibition in 14 women with PMDD and 13 healthy controls [44]. Participants pressed a key in response to sequential single letters, but inhibited their response when the same letter appeared twice in a row. In a whole-brain analysis (uncorrected), women with PMDD displayed lower activity than controls during both phases of the menstrual cycle in task-related parietal areas. Region of interest analyses of the amygdala and insula showed a cluster of voxels in the left insula that had more activity in the PMDD subjects during the luteal than the follicular phase, whereas controls showed no effect of menstrual phase, producing a significant group by phase interaction. No cerebellar results were reported.

Amygdala reactivity was contrasted in women with and without PMDD by pairing fMRI with an emotional face-matching task [45]. Contrary to expectation, the PMDD group had higher bilateral amygdala reactivity than the control group in the follicular, but not the luteal phase. Women with PMDD who also had high trait anxiety, however, did display higher right amygdala activation in the luteal as compared to the follicular phase. The control group showed the same menstrual cycle effect in the left amygdala. In women with PMDD, bilateral amygdala reactivity in the follicular phase was positively correlated with serum progesterone levels and right amygdala reactivity in the luteal phase was correlated with self-rated anxiety and depression. As compared to the control group, amygdala reactivity in women with PMDD habituated more across sessions. The authors concluded that trait anxiety and progesterone levels modulate menstrual cycle related amygdala reactivity in women with PMDD. Regions outside the amygdala were not assessed.

In another fMRI study, women with and without PMDD were evaluated during valence-cued anticipation and presentation of emotional images in 14 regions of interest in both hemispheres [46]: amygdala, anterior cingulate, insula, Brodmann Area [BA] 6, 8, 9 and 10. No results were reported from exploratory whole-brain analyses (uncorrected). In women with PMDD, progesterone levels were correlated with the response to presentation of positive emotional images in the dorsolateral prefrontal cortex. During color-cued anticipation of negative images in the luteal phase, women with PMDD had greater activation in the medial and dorsolateral prefrontal cortex than in the follicular phase, and more than control women in the luteal phase, suggesting that cortical emotional reactivity during anticipation may be important in PMDD.

In the above-mentioned study of Baller and associates, the working memory task generated greater activation in women with PMDD than controls, assessed with fMRI across the one asymptomatic and the two symptomatic hormone conditions, in 8 regional clusters [47]. The largest cluster was in the superior frontal gyrus (BA8). Task-related activity in PMDD patients was correlated with psychosocial/somatic impairment in 11 regional clusters that overlapped with the areas of greater activation in PMDD, particularly in frontal and parietal cortices. Although the cerebellum contained clusters with significantly greater activation in PMDD patients than controls, and correlation of activation with impairment in PMDD patients, the cerebellar clusters represented only 3% and 1% of the total number of voxels showing these effects, respectively. It is important to note, however, that all of the cerebellar results were confined to cerebellar lobules VI and VII, the “emotional” areas of the cerebellum [21].

Across the fMRI studies, the most consistent abnormality associated with premenstrual dysphoria was greater frontal activation, particularly in the dorsolateral prefrontal cortex, in PMDD patients as compared to controls. This was measured when patients were symptomatic in response to affectively negative words [43], or anticipation of negative images [46], and during an affectively neutral working memory task assessed across one asymptomatic and two symptomatic conditions [47], although not during an easier affectively neutral Go/NoGo task [44].

### 3.5. Measures of brain structure

As part of one of the MRS studies of women with (*n* = 12) and without (*n* = 13) PMDD, tissue composition in three compartments (gray matter, white matter and cerebrospinal fluid) was compared in the two groups within medial prefrontal cortex using a double-inversion recovery one-dimensional projection method [57]. Although there were no significant group differences in MRS measures described above, women with PMDD had a lower percentage of gray-matter than controls in medial prefrontal cortex [42].

More recently, optimized voxel-based morphometry (VBM) of high-resolution structural MRI scans was used to compare regional gray matter in the brains of 15 PMDD patients and 15 healthy women [48]. The PMDD group had lower gray-matter density in a cluster in the left parahippocampal gyrus, and higher gray matter density than control women in a larger cluster in the left hippocampal gyrus. Neither effect was related to PMDD symptom severity. Although differences in the cerebellum were not reported, the lower left panel of their Figure 2 [48], suggests that the area of higher gray-matter density in PMDD patients may extend into nearby cerebellar lobules V and VI, the latter being consistently implicated in emotional processing [21].

Our group also has used optimized VBM with structural MRI to study PMDD [49]. The only whole-brain corrected difference between 12 women with PMDD and 13 healthy controls was a large cluster in the posterior cerebellum where women with PMDD had higher gray-matter volume than control women. Virtually the same cluster exhibited a decrease in gray-matter volume with advancing age in the control group, but not in the PMDD group. After a median split on participant’s age, the difference in gray-matter volume between the groups remained significant for women older but not younger than 30. Group differences in gray-matter volume as well as the effect of age on gray-matter volume included over four times as much of the cerebellar vermis as the cerebellar hemispheres. Region-of-interest analyses for both effects using a standard cerebellar partitioning scheme [58] revealed significant peak voxels within all six partitions previously associated with emotional processing in a meta-analysis [21], but few significant peaks in the other 12 partitions. We concluded that PMDD is associated with reduced age-related gray-matter loss in the “emotional” cerebellum. Considering this finding in the context of fMRI results, it is notable that alterations in gray-matter structure can influence BOLD signal [59], possibly confounding effects noted with fMRI. Moreover, the difference in the age effect on cerebellar structure suggests that symptom and/or disease severity may not have been correlated with this structural feature of the brain in PMDD because the structural differences derives from age-related changes in the healthy controls but not those with PMDD.

There are several possible reasons for different results of the two structural brain studies. Notably both studies were of small sample size, and Jeong and associates used a version of the VBM software (VBM2), which did not include the high-dimensional DARTEL normalization designed to improve registration in the version we used (VBM8). This may be important because prior procedures for normalization to whole-brain MRI templates produce inaccuracies especially in the cerebellum [58,60]. Jeong and associates acquired MRI data in the luteal phase, whereas we scanned in both the follicular and luteal phases. Notably, although the participant ages in the comparison groups were equivalent in both studies, Jeong and associates used age as a covariate of no interest, a manipulation that could reduce gray-matter differences allocated to the grouping variable by allocating some of the variance to the age variable. When we reanalyzed our data using participant age as a covariate of no interest, the cluster of higher gray-matter volume in women with PMDD than in healthy controls was 14% smaller than in the original analysis.

### 3.6. What mechanisms could explain preserved gray matter during aging in PMDD?

The finding of preserved cerebellar structure with aging in PMDD warrants replication, but has some interesting implications which will now be discussed. The aforementioned whole-brain structural studies found a greater proportion of voxels with higher rather than lower gray matter in women with PMDD than in healthy controls (67% and 100%, respectively in the results of Jeong and associates and Berman and associates) [48,49]. This observation adds to the evidence differentiating PMDD from other psychiatric disorders with dysphoric symptoms, which are generally associated with volume deficits, especially in the cerebellum. A few studies found no difference, but more often studies reported abnormally low cerebellar gray matter in samples with PTSD [61,62], major depressive disorder [63,64], bipolar disorder [61,65], anxiety disorder [62] and depressive and anxiety-related personality traits in a healthy sample [66].

A positive correlation between the duration of bipolar illness and the degree of bilateral cerebellar GM deficits was observed in medication-naive patients [65], and one study found higher gray-matter volume of lobule VII in both left and right cerebellar hemispheres of first-episode bipolar patients [67]. An enlarged vermis has also been shown in monkeys after a period in which the animals were subjected to stress [68]. These results support the view that relative cerebellar hyperactivity may be a compensatory response to stress, leading to later volume losses only when symptoms of affective disorder are overly severe and/or prolonged [61]. Since increases in local activity as brief as a few hours have been reported to increase local gray-matter volume [69,70], chronic but remitting periods of stress could thus produce progressive increases in cerebellar gray matter, or could counteract age-related gray-matter loss.

Because cognitive complaints are common in PMDD, but have been difficult to quantify, we suggested that most women with PMDD exert greater effort, or other compensatory mechanisms that increase cerebellar activity, in order to maintain cognitive functioning during symptomatic periods, which can total over 3000 days over the lifetime [49]. Since regions involved in cognitive and emotional processing overlap anatomically in the cerebellum, these compensatory mechanisms could act as mental “exercises” that preserve structure in the cognitive/emotional portions of the cerebellum during aging in the same way that running with leg weights can preserve muscle structure. The findings discussed above, showing that preserved cerebellar structure is one of the best predictors of preserved cognitive function with advanced age [28,33], suggest the intriguing possibility of better cognitive aging in women with PMDD. Confirmation of this testable notion could inform a previously proposed strategy [32] of using cerebellar plasticity in interventions designed to improve cognitive aging.

Cerebellar hyperactivity also may contribute to the higher serum levels of several neuroprotective factors that have been reported in women with PMDD relative to healthy controls [71,72], and enhanced neuroprotection could in turn preserve cerebellar gray matter during aging. A recent investigation of two neuroprotective factors found that women with PMDD assessed during the luteal phase had higher serum levels than healthy women of the important neurotrophin, brain-derived neurotrophic factor (BDNF), and higher levels of a key member of the stress-protective molecular chaperone system, heat-shock protein-70 [71].

The cerebellum also contains the highest density of brain receptors for the neuroprotective hormone leptin [73]. Leptin has centrally mediated regulatory effects on food intake, energy expenditure, body mass, and reproduction, with increased plasma concentrations in the late follicular and luteal phases of normal cycles [74]. Cerebellar gray-matter volume has been correlated with plasma leptin concentration among older adults [75], and cerebellar gray-matter volume was increased when we gave genetically-deficient adults daily leptin supplements [76], but cerebellar gray-matter volume reversibly decreased when leptin supplements were withheld for a month [77].

Woman with PMS (*n* = 21) were reported to have higher plasma leptin than healthy subjects (*n* = 22) in both follicular (*P* < 0.001) and to a lesser extent luteal (*P* < 0.08) assessments [72]. Although follicular-phase leptin was not independently different from healthy women in the two subsequent studies that have been conducted, participants with premenstrual dysphoria had 16% [78] and 36% [79] higher leptin levels than control women, suggesting overall support for the original finding. In contrast, low leptin levels have been associated with major depression and suicide [80,81], depressive and anxious states [82], depressive symptoms [83,84] and resistance to antidepressant treatment [85]. Low leptin in four independent studies of Alzheimer’s Disease [86] and in patients with mild cognitive impairment [87] support the suggestion that leptin may be beneficial for age-related cognitive decline [86]. The high density of leptin receptors in the cerebellum and the ability of leptin supplementation to increase cerebellar gray matter reversibly, combined with reports of higher plasma levels of leptin in women with premenstrual dysphoria and with reports of linkage of higher leptin levels to better cognitive aging, which is itself linked to cerebellar gray matter, make leptin a promising candidate for mediating preserved cerebellar gray matter with aging in women with PMDD.

An additional possibility is that just as PMDD symptoms represent an abnormal response to normal fluctuations in ovarian steroids [56,88], neuroprotective effects of these steroids in premenopausal and perimenopausal women [89,90] could be enhanced in women with PMDD. Estrogen is a potent energy regulator, with overlap in function and neural targets with leptin that has been described as “overwhelming” [91]. Recent research has shown the cerebellum to be an important target for estrogen, where locally synthesized estradiol modulates glutamatergic neurotransmission and has been implicated in neuroprotection from many diseases both in animal models and human subjects [92]. Estrogen therapy that is started around the time of menopause has been shown to preserve both cognition and gray-matter structure, particularly in the cerebellum [93,94,95]. In a VBM study where whole-brain corrected cluster sizes were provided, the cerebellum contained 86% of brain voxels where gray matter volume was preserved in patients receiving estrogen replacement [94].

## 4. Summary of brain imaging results

Table 1 shows that most imaging studies of PMDD have assessed only one or a few brain regions. Most of the reported regional differences between women with and without PMDD came from a single study, or are inconsistent. The most consistent findings are greater relative activity in the dorsolateral prefrontal cortex and posterior lobules VI and VII of the neocerebellum, as detailed below.

What is the evidence for a dorsolateral prefrontal cortex role in premenstrual dysphoria? Measurement of both rCBF and the BOLD signal indicated greater dorsolateral prefrontal activation during a working memory task in women with PMDD than controls, correlated with measures of PMDD impairment and duration [47]. Women with PMDD also had greater luteal-phase BOLD response in the dorsolateral prefrontal cortex than controls during cued anticipation of negative emotional images, and their response in dorsolateral prefrontal cortex to positive images was correlated with serum progesterone levels [46]. Phase-dependent changes in binding of the serotonin precursor in the dorsolateral prefrontal cortex were saliently correlated with concomitant change in the affective symptoms of PMDD [41].

What is the evidence for a cerebellar role in premenstrual dysphoria? Early imaging studies of premenstrual dysphoria did not assess the cerebellum or used it as a reference region. Because of the long dominant view that the cerebellum was primarily important for motor function, these unfortunate practices are common in neuroimaging research.

One principled way to determine what brain regions have abnormalities associated with PMDD is comparing results from whole-brain analyses that were statistically corrected for multiple comparisons. There have been two such structural studies using VBM, two PET activation studies and two fMRI activation studies (Table 1). Greater activity within dorsolateral prefrontal cortex was associated with PMDD in one PET study [47] and one fMRI study [47]. Greater cerebellar activity was observed in one of the fMRI studies [47], both PET studies [47,54], and higher cerebellar grey matter volume in PMDD patients as compared to controls, which could contribute to measures of greater activation, was reported in one of the two structural studies [49]. All these results were localized to the part of the cerebellum that has been consistently associated with emotional processing [21], and no study reported reduced cerebellar activity or structure in groups with PMDD as compared with controls, increasing confidence that over-activation of the “emotional” cerebellum is involved in the pathophysiology of chronic premenstrual dysphoria.

An interesting question is why cerebellar abnormalities in PMDD are more prominent for PET-assessed measures of activation, where they represent the largest and strongest differences in both glucose metabolism and blood flow, as compared to abnormalities accessed via the BOLD signal, a more indirect measure of neural activity that depends on neurovascular coupling. The origins of the BOLD fMRI response are complex, and not completely understood [96], but by middle adulthood vascular aging decreases the sensitivity of the signal to neural activity [97,98,99], with large variance across individuals and brain regions. Coupled with evidence that cerebellar dysfunction plays an important role in vascular dementia [100], and the large structural differences between cerebellar and cerebral cortex (for example; 80% *vs* 50% gray matter), this suggests the possibility of different age trajectories for the ability of BOLD measurement to index neural activity in the two structures.

This notion is supported by studies of age-related variability in the moment-to-moment BOLD signal. The mean BOLD signal is currently the most utilized measure of brain function in neuroimaging, including all of the fMRI studies reviewed here. It has been reported, however, that over five times as much of the variability in participants’ age can be predicted by the standard deviation of the BOLD signal (BOLD-SD) than can be predicted by the mean, and that BOLD-SD is also a better predictor than BOLD-mean of performance on cognitive tasks [101,102]. In the cerebral cortex, greater BOLD-SD, as with greater BOLD-mean, is generally associated with younger age and faster more consistent task performance, but in the cerebellum increased BOLD-SD is associated with older age and slower less consistent task performance [101]. The authors speculate that greater variability with age could reflect compensatory processes serving to counteract reduced network complexity and integration or could represent a dysfunctional form of variability [101].

Combined with strong PET evidence for greater activation of “emotional” cerebellum in women with PMDD than controls [47,54], sparse evidence from similar fMRI studies, and evidence that PMDD patients have gray matter-preservation with aging in the neocerebellum [49], the BOLD variability results suggest that the standard fMRI BOLD-mean measure may be insensitive to activation differences that differentiate relatively older (> age 30) women with and without PMDD. Within the densely vascularized cerebellum, the BOLD-mean measure both diminishes and becomes more variable with age. In contrast, the decreased BOLD variability with aging in the cerebral cortex would offset the decreased mean signal, relatively preserving sensitivity to activation differences between groups with and without PMDD.

## 5. Conclusions

Taken as a whole, brain imaging studies best support the hypothesis that premenstrual dysphoria is associated with increased activity of the dorsolateral prefrontal cortex and lobules VI and VII of the posterior cerebellum. Both brain regions have been associated with emotional processing and mood disorders, working memory and executive functions. Combined with the above discussed recent expansion of current understanding of cerebellar physiology to include the modulation and fine-tuning of all forms of brain computation, this suggests that increased activity within these two regions in PMDD patients probably represents coactivation of both regions within a cerebro-cerebellar feedback loop regulating emotional and cognitive processing. Future brain imaging studies of premenstrual dysphoria should explore the functioning of this cerebro-cerebellar circuit.

## Figures and Tables

**Table 1:** Brain imaging studies of premenstrual dysphoric disorder and premenstrual syndrome.

Author/Year	Modality	Subjects	Brain Imaging Measures	Major Imaging Results	Were findings reported in cerebellum?
Buchpiguel et al. 2000 [37]	[^99m^Tc]HMPAO SPECT	7 PMS, 7 control	Regional cerebral blood flow in frontal, temporal and parieto-occipital cortices, thalamus, and basal ganglia, normalized to cerebellum	From follicular to luteal phase, temporal lobe blood flow decreased in PMS patients, more than in controls. The degree of decrease was correlated with the level of depression	Not assessed *per se*; used as reference region
Rasgon et al. 2001 [38]	^1^H-MRS	5 PMDD, 6 control	Ratios of myo-inositol, choline, and NAA (N-acetyl-aspartate) to creatine within medial prefrontal and occipito-parietal cortex	Changes in NAA (medial prefrontal cortex, cingulate gyrus) and choline (parietal white matter) with menstrual phase, but no significant group differences	Not Assessed
Epperson et al. 2002 [39]	1H-MRS	8 PMDD, 12 control	GABA in midline occipital cortex	From follicular to luteal phase, occipital GABA decreased in controls but increased in PMDD women to higher levels than controls	Not assessed *per se*; used as reference region
Jovanovic et al. 2006 [40]	[^11^C]WAY-100635 PET	5 PMDD, 5 control	5-HT1a receptors in six brain regions : DLPFC, OFC, ACC, amygdala hippocampus and dorsal raphe	From follicular to luteal phase, 5-HT1a binding in the dorsal raphe increased more in controls than in women with PMDD	Not Assessed
Eriksson et al. 2006 [41]	[^11^C]-5-hydroxy tryptophan PET	8 PMDD	Serotonin precursor trapping in whole brain (cumulative) and bilateral regions of interest: DLPFC, MPFC, putamen and caudate	Phase-dependent change in trapping of the serotonin precursor were correlated with concomitant change in affective, but not physical PMDD symptoms in whole brain and most assessed regions, particularly dorsolateral prefrontal cortex	Not Assessed
Batra et al., 2008 [42]	MRS	12 PMDD, 13 control	Ratio of glutamate to creatinine and tissue composition in medial prefrontal cortex	No significant group difference in glutamate, but medial prefrontal cortex had a lower percentage of GM in women with PMDD than control women	Not Assessed
Protopopescu et al. 2008 [43]	fMRI	8 PMDD, 12 control	BOLD activation during an emotional word Go/NoGo task in whole-brain (corrected) and in selected regions: frontal cortex, amygdala and nucleus accumbens	Symptomatic women with PMDD had higher amygdala activation by negative words and lower nucleus accumbens activation by positive words than controls	No
Rapkin et al. 2011 [3]	FDG PET	12 PMDD, 12 control	Regional glucose metabolism during an affectively neutral vigilance task in the whole-brain (corrected)	From follicular to luteal phase, woman with PMDD, but not controls, had increased cerebellar metabolism correlated with worsening of mood	Yes
Bannbers 2012. [44]	fMRI	14 PMDD, 13 control	BOLD activation during response inhibition in an affectively neutral Go/NoGo task in whole-brain (uncorrected) and amygdala and insula ROIs	From follicular to luteal phase, left insula activity increased in PMDD patients but not controls. Women with PMDD has less activity than controls in task-related parietal areas	No
Gingnell et al. 2012 [45]	fMRI	14 PMDD, 15 control	BOLD activation during an emotional face-matching task in the amygdala	In the follicular phase, women with PMDD had enhanced amygdala reactivity, correlated with serum progesterone. In the luteal phase, controls and PMDD women with high trait anxiety had enhanced amygdala reactivity that was correlated with anxiety/depression in PMDD patients	Not Assessed
Gingnell et al. 2013. [46]	fMRI	14 PMDD, 14 control	BOLD activation by emotional images and valence-cued anticipation in whole-brain (uncorrected) and ROIs: amygdala, ACC insula, BA 6, 8, 9 and 10	During luteal-phase anticipation of negative images, women with PMDD had more medial and dorsolateral prefrontal cortex activation than in the follicular phase, or than control women had in the luteal phase. Progesterone levels were correlated with dorsolateral prefrontal cortex response to positive emotional images in women with PMDD	No
Baller et al. 2013. [47]	O-15 PET	15 PMDD, 15 control	Whole-brain (corrected) rCBF during an affectively neutral 2-back working memory task	Compared to controls, woman with PMDD had greater activity in 9 regional clusters and activity was correlated with psychosocial/somatic impairment in overlapping clusters, particularly in cerebellum and frontal cortex. PMDD duration and age of onset also correlated with activation and over-activation in PMDD bilaterally within both structures	Yes
	fMRI	14 PMDD, 14 control	Whole-brain (corrected) BOLD activation during an affectively neutral 2-back working memory task	Mostly agreed with PET results. Compared to controls, woman with PMDD had greater activity in 8 regional clusters and activity was correlated with psychosocial/somatic impairment in overlapping clusters, particularly in frontal and parietal cortex but also including the cerebellum. PMDD age of onset also correlated with activation within these structures	Yes
Jeong et al. 2012. [48]	VBM	15 PMDD, 15 control	Whole-brain (corrected) GM density one time during luteal phase	Compared to controls, woman with PMDD had lower GM in the left parahippocampal gyrus and higher GM in a larger cluster in the left hippocampal gyrus	No
Berman et al. 2013. [49]	VBM	12 PMDD, 13 control	Whole-brain (corrected) GM volume one time during luteal or follicular phase	Compared to controls, woman with PMDD, particularly those over age 30, had greater GM in posterior "emotional" cerebellum, and the same areas did not exhibit the age-related GM loss seen in controls	Yes
